# Multi-system manifestations of *Mycoplasma pneumoniae* infection in a young patient

**DOI:** 10.1099/jmmcr.0.005117

**Published:** 2017-09-19

**Authors:** Ibrahim Al Busaidi, Mohammed Al-Amin, Shadin Ibrahim, Abdullah Balkhair, Zied Gaifer

**Affiliations:** Infectious Diseases Unit, Department of Medicine, Sultan Qaboos University Hospital, Muscat, Oman

**Keywords:** *Mycoplasma pneumoniae*, extrapulmonary manifestations, erythema multiforme, myringitis, ITP

## Abstract

**Introduction.** *Mycoplasma pneumoniae* is a small cell-wall-lacking bacterium that belongs to the mycoplasma (Mollicutes) prokaryote micro-organisms. It is a common cause of both upper and lower respiratory tract infections in all age groups. Respiratory illness is the most common manifestation of *M. pneumoniae* infection; however, extrapulmonary involvement may be present or predominant. The skin, mucus membranes, central nervous system, cardiovascular system, haematopoietic system, kidneys and musculoskeletal system are the most commonly involved extrapulmonary sites. Immune thrombocytopenia purpura has been reported as a rare haematological manifestation of mycoplasma infection. Here, we report, with a literature review, the case of a young adult with *M. pneumoniae* infection, presenting with acute febrile illness, myringitis, erythema multiforme, mild Raynaud’s phenomenon symptoms and severe thrombocytopenia.

**Case presentation.** Our patient was a 24-year-old healthy man who presented to an emergency department with acute febrile illness, upper respiratory tract infection symptoms, myringitis, erythema multiforme skin lesions, severe thrombocytopenia, and pale and cold hands. *Mycoplasma* serology suggested acute *M. pneumoniae* infection. The patient had a complete resolution of symptoms and gradual recovery from the thrombocytopenia after a course of anti-*Mycoplasma* therapy with azithromycin. Our case illustrates the multi-system involvement of *M. pneumoniae* infection.

**Conclusion.** *M. pneumoniae* is a frequent cause of upper and lower respiratory tract infections in children and young adults. Multi-system involvement including the skin, vascular and haematological systems in young adults with upper or lower respiratory tract infection, as in our patient, should raise the suspicion of *Mycoplasma* infection. Our case also illustrates an excellent clinical response and recovery from thrombocytopenia shortly after anti-*Mycoplasma* antimicrobial therapy.

## Abbreviation

ITP, immune thrombocytopenia purpura.

## Introduction

*Mycoplasma pneumoniae* is one of the smallest ubiquitous self-replicating organisms that lack a cell wall [1]. It was first isolated and named after its discovery in sputum in tissue culture from a patient with atypical pneumonia in 1944 [2]. Infection is primarily seen in childhood and adolescence in the respiratory tract, with the peak incidence at between 5 and 15 years of age. *M. pneumoniae* was reported to represent 15–20 % of cases of community acquired pneumonia in one study [3]. Respiratory tract infections are usually preceded with gradual onset malaise, pharyngitis and sinus congestion, and are occasionally associated with otitis media and myringitis. The infection has an incubation period of 6–32 days, and it is probably transmitted by droplet inhalation and direct contact with an infected person [4]. A wide variety of extrapulmonary manifestations have been described in association with *M. pneumoniae* infection (Table 1) [5, 6]. The possible mechanisms for these manifestations are a direct effect of *M. pneumoniae*, immune-mediated effects and vascular occlusion [5].

**Table 1.** Common extrapulmonary manifestations of *M. pneumoniae* infection

**System**	**Clinical manifestation**	**System**	**Clinical manifestation**
Cardiovascular system	Pericarditis Endocarditis Myocarditis Kawasaki disease Cardiac thrombus Aortic thrombus	Nervous system	Encephalitis/cerebellitis Myelitis Aseptic meningitis Guillain–Barré syndrome Neuropathy Acute cerebellar ataxia Opsoclonus-myoclonus syndrome Stroke Acute disseminated encephalomyelitis Psychological disorders
Dermatological	Erythema multiforme Urticaria Anaphylactoid purpura Erythema nodosum Cutaneous leukocytoclastic vasculitis Stevens–Johnson syndrome Mucositis Subcorneal pustular dermatosis	Genitourinary system	Glomerulonephritis IgA nephropathy Renal artery embolism Priapism
Haematological/ haematopoietic system	Cold agglutinin formation Haemolytic anaemia Haemophagocytic syndrome Thrombocytopenic purpura Infectious mononucleosis Disseminated intravascular coagulation	Gastrointestinal	Hepatitis Pancreatitis

Serology using enzyme immunoassays is the most widely used test for the diagnosis of *M. pneumoniae* infection. IgM and IgA antibodies both appear in the early phase of infection and detection of any or both antibodies indicates an acute infection [7]. Diagnosis can be also made using a single titre of IgM greater than or equal to 1 : 64 or either seroconversion or a threefold rise in the titre of IgG. Detection of *M. pneumoniae*-specific antigens has a sensitivity and specificity of 91.3 and 100 %, respectively [1]. The PCR for detection of *M. pneumoniae* in clinical samples has a 73 to 92 % sensitivity, and a 96 to 100 % specificity. PCR has a better diagnostic yield for sputum compared with throat and nasopharyngeal swabs [8].

## Case report

A 24-year-old previously healthy Indian man presented to the emergency department at Sultan Qaboos University Hospital (Muscat, Oman) with acute febrile illness for 4 days, associated with sore throat, mild cough, right ear pain, mild nose bleed and skin rash involving the extremities. The target-like lesions started 1 day after the fever in the upper and then the lower extremities over 24 h. The patient also had pale and cold hands on presentation. He worked as an accountant in a company and he had been in Oman for almost 12 months. He had no recent travel history or significant exposures. He was not taking regular medications. His vaccination history was up to date.

**Fig. 1.**
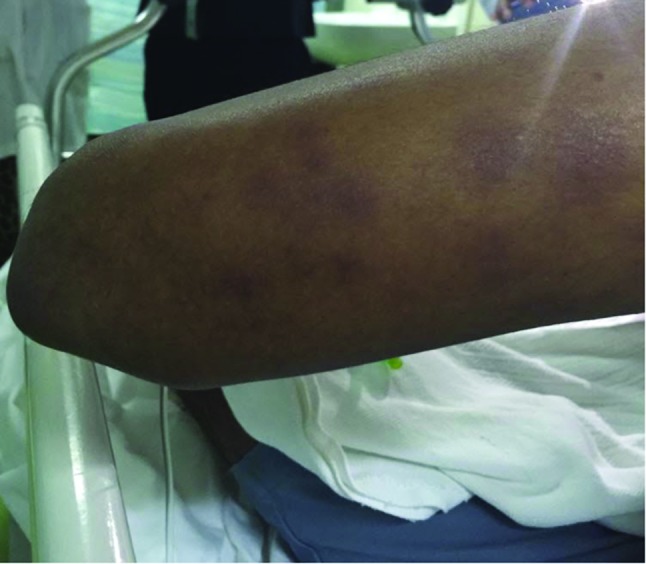
Purple-coloured, targets like lesions with central clearing on the posterior aspect of the left arm.

On initial assessment in the emergency department, he appeared well, alert and conscious. He was febrile (38.1°C) and tachycardic (heart rate 100 beat min^−1^). His blood pressure was 149/76 mmHg, his respiratory rate 20 breaths min^−1^ and his oxygen saturation was 98 % on room air. General examination showed no pallor, icterus or conjunctival haemorrhages, but there was right anterior cervical lymph node enlargement (1.5×1.0 cm). Ear, nose and throat examination revealed a congested oropharynx and right-sided myringitis. Skin examination showed raised purple-coloured, target-like lesions with central clearing consistent with erythema multiforme involving the upper and lower limbs (Fig. 1). The patient's hands were pale and cold compared with the rest of the upper limbs.

Initial investigations revealed severe thrombocytopenia (3×10^10^ platelets l^−1^), normal haemoglobin (12.7 g dl^−1^), normal haematocrit (35 %) and a total white cell count of 2.8×10^9^ cells l^−1^ with mild lymphopenia (absolute lymphocyte count of 1.0×10^9^ cells l^−1^). The patient's liver function panel was normal: alanine aminotransferase (36 U l^−1^), aspartate aminotransferase (54 U l^−1^), albumin (41 g l^−1^) and bilirubin (15 µmol l^−1^). His renal function and electrolytes panel was normal. A blood film for malaria screening was negative and a chest X-ray was normal. Epstein–Barr virus, cytomegalovirus and human immunodeficiency virus serology were all negative. A respiratory virus screen was negative. *M. pneumoniae* serology was consistent with acute infection, with both *Mycoplasm*a IgA and IgG positive, and negative IgM. Based on the clinical presentation and positive serology, the diagnosis of acute *M. pneumoniae* with multiple extrapulmonary manifestations was made. The patient was treated with a 7 day course of oral azithromycin at a dose of 500 mg daily with excellent clinical response. The course of antibiotic was well tolerated and no adverse reactions were reported.

## Discussion

Our case illustrates the extrapulmonary multi-system involvement of *M. pneumoniae* infection, including the ear, skin and haematological and vascular systems. *M. pneumoniae* causes otitis media and (non-bullous or bullous) myringitis. Historically, *M. pneumonia*e was described as causing bullous myringitis characterized by the presence of blistering or vesicles on the tympanic membrane [9]. Recent data suggests that bullous myringitis is not specific for *Mycoplasma* and it is caused by common organisms of otitis media. Cutaneous lesions have been reported in 25–33 % of patients with *M. pneumoniae* infection [10]. A wide array of skin manifestations has been reported regardless of the stage and location of the infection. The most common of these is the exanthematous or ‘maculopapular’ eruption [6]. Our patient had erythema multiforme, which is a common form of this type of skin lesion. Stevens–Johnson syndrome is a severe form of erythema multiforme and it represents 1–5 % of cutaneous disease [11].

Thrombocytopenia is not a common manifestation of *M. pneumoniae* infection and it may occur in the setting of thrombotic thrombocytopenic purpura and disseminated intravascular coagulation [2]. Aviner and colleagues described eight cases of *M. pneumoniae*-related immune thrombocytopenia purpura (ITP) that occurred concomitantly with the infection [12]. In those cases, the patients' ages ranged from 7 months to 44 years and platelet counts ranged from 2 to 66×10^3^ platelets µl^−1^. Compared with classical ITP, a more severe course and bleeding were observed in those cases, requiring corticosteroids and intravenous immunoglobulins. The possible mechanisms of *M. pneumoniae-*induced ITP are immune complex induced platelet aggregation and direct destruction of platelets [13]. Our patient had a mild form of the disease with minimal self-limited mucocutaneous bleeding and his platelets increased gradually shortly after antibiotic therapy was initiated.

Cold agglutinins are IgM antibodies produced during the early phase of *M. pneumoniae* infection, and they are responsible for some haematological and vascular manifestations including haemolytic anaemia, Raynaud’s phenomenon, vascular ischaemia and renal failure. Our patient had pale and cold hands likely due to cold-agglutinin-induced vasospasm, which has been described in the literature as ‘cold hands’ [14]. Symptoms resolved after antibiotic therapy and no vascular complications were reported in our patient.

### Conclusion

*M. pneumonia* is a frequent cause of upper and lower respiratory tract infections in children and young adults. A variety of extrapulmonary diseases are caused by *M. pneumoniae* that a clinician should be aware of. Our patient had multi-system involvement, including the ear, skin, and vascular and haematological systems. *M. pneumoniae* should be considered as a cause of ITP in children and young adults in the appropriate clinical setting. *M. pneumoniae-*induced ITP can be more severe compared to classic ITP. Cold-agglutinin-induced vasoconstriction is one manifestation and possibly the mildest form of a wide spectrum of vascular complications of *M. pneumoniae* infection. Early initiation of anti-mycoplasma therapy is essential to eliminate the trigger and enhance the resolution of associated manifestations.
